# Too Little, Too Late? Redefining the Legacy of HMAS *Perth* (I), an Australian Warship Sunk in Indonesian Waters

**DOI:** 10.1007/s41636-023-00401-7

**Published:** 2023-05-26

**Authors:** Natali Pearson

**Affiliations:** Sydney Southeast Asia Centre, University of Sydney, Room 638, Brennan-MacCallum Building (A18), Camperdown, NSW 2006 Australia

**Keywords:** Indonesia, Australia, shipwreck, community

## Abstract

Australian warship HMAS *Perth* (I) was sunk during the Battle of the Sunda Strait in 1942, claiming the lives of 353 men. It was not until 2017 that Indonesian and Australian authorities conducted a joint archaeological survey of the site. They found that *Perth* had been salvaged on an industrial scale, with less than 40% of the vessel remaining. The discovery devastated those with an emotional connection to *Perth*, and, following strong Australian government advocacy, informed Indonesia’s decision to establish a maritime conservation zone, the nation’s first, around the site. Although the 80 years since *Perth* sank have been characterized by a lack of official engagement, this article proposes that the recent destruction of *Perth* is not the end, but the beginning, of a new era of bilateral cooperation, founded on the recognition that the wreck has historical significance for Australia as well as potential benefits for local communities in Indonesia.

## Resumen

El buque de guerra australiano *HMAS Perth* (I) fue hundido durante la batalla del estrecho de Sunda en 1942, segando la vida de 353 hombres. No fue sino hasta 2017 que las autoridades de Indonesia y Australia realizaron un estudio arqueológico conjunto del sitio. Descubrieron que el *Perth* había sido objeto de un salvamiento a escala industrial, quedando menos del 40% del buque. El descubrimiento devastó a quienes tenían una conexión emocional con el *Perth* y, luego de una fuerte defensa por parte del gobierno australiano, eso influyó en la decisión de Indonesia de establecer una zona de conservación marítima, la primera del país, alrededor del sitio. Aunque los 80 años desde que el Perth se hundió se han caracterizado por la falta de compromiso oficial, este artículo propone que la reciente destrucción del *Perth* no es el final, sino el comienzo de una nueva era de cooperación bilateral, basada en el reconocimiento de que el naufragio tiene un significado histórico para Australia, así como beneficios potenciales para las comunidades locales en Indonesia.

## Résumé

Le navire de guerre australien *HMAS Perth* (I) a sombré au cours de la Bataille du Détroit de Sunda en 1942, ayant entraîné la mort de 353 hommes. Ce n'est qu'en 2017 que les autorités indonésiennes et australiennes ont conduit une étude archéologique conjointe du site. Ils ont découvert que le *Perth* avait fait l'objet d'une récupération à une échelle industrielle, moins de 40 % du navire y ayant échappé. La découverte a bouleversé ceux qui avaient un lien sentimental avec le *Perth* et, à la suite d'initiatives importantes de soutien du gouvernement australien, elle a pesé sur la décision de l'Indonésie d'établir une zone de conservation maritime autour du site, la première de la nation. Bien que les 80 années écoulées depuis le naufrage du *Perth* se soient caractérisées par une absence d'engagement officiel, cet article postule que la destruction récente du *Perth* n'est pas la fin, mais le début d'une nouvelle ère de coopération bilatérale, fondée sur la reconnaissance que l'épave a une signification historique pour l'Australie ainsi que des bénéfices potentiels pour les communautés locales en Indonésie.

## Introduction

The year 2022 marked 80 years since Australian warship HMAS *Perth* (I) was sunk by Japanese gunfire and torpedoes in the Battle of the Sunda Strait (28 February–1 March 1942). More than half of *Perth*’s company went down with their ship, which rests in the waters of Indonesia’s Banten Bay, and many of the survivors went on to endure significant hardship and suffering as prisoners of war. The poignancy of this 80th anniversary, already a solemn occasion for Australian survivor and descendant communities, was exacerbated by the knowledge that very little of *Perth* remains. Although the wreck has been subject to ongoing human intervention since its rediscovery in the 1960s, most of the destruction has taken place in the last decade. Quite distinct from the smaller scale and largely opportunistic salvaging and souveniring of previous decades, this more recent salvaging has been conducted at speed and at an industrial level.

The devastation and desecration of *Perth* has naturally prompted questions within both Australia, as flag state, and Indonesia, as coastal state, that seek to apportion responsibility and develop “lessons learned” to prevent such scenarios in the future. But, as this article proposes, the loss of *Perth*—in 1942 and subsequently—also raises broader questions about shipwreck legacies, communities, and justice. How do we understand a shipwreck’s legacy, and does that change when the site itself is gone? Can we conceptualize a wreck’s communities in a way that both recognizes, with sensitivity, survivors and descendants and also makes space for those who have lived with the wreck in their waters for far longer than the vessel ever saw active service? Is it possible to account for loss in a way that does justice to these communities, and how might this affect our thinking about shipwreck management for and in the future?

This article seeks to intervene in the narrative of loss that dominates discussions about *Perth*—and that leads to stigmatization and blame—and to do so with both sensitivity and hope. Yet it does not seek to minimize or detract from the intense suffering and anger that have accompanied the subsequent destruction of the wreck; these responses are appropriate. It does, however, ask whether the legacy of a shipwreck is necessarily defined by its material presence, and how this might change when that presence has been partially—or, as has been the case with other naval shipwrecks in Indonesian waters, entirely—destroyed. In doing so, this article draws less on approaches that prioritize preservation, the law, or international relationships (Browne 2019; Forrest 2019; Lin 2020; Manders et al. 2021). Instead, it focuses on emerging and critical perspectives, drawing attention to the “insistence on intervention” (Rich 2021:227) that pervades archaeological approaches and the extent to which scientists (and scholars) are positioned as saviors within this paradigm. Rather than sites to be explored and saved, shipwrecks like *Perth* can be places ideally suited for testing and even extending theoretical limits (Rich 2021)—for reflecting on what it is we want from them and what they can tell us about ourselves.

An overview of *Perth*’s service history sets our scene. Considering *Perth*’s history in detail, as I do here, allows for a stronger appreciation of the regard and pride with which this ship was held during both its peacetime and wartime service, as does the use of newly available historical data including crew diaries and photographs. The article then turns more fully to *Perth*’s presence as both wreck and—over time—reef in Indonesian waters, and the beginnings of its “death by a thousand cuts” (Hosty et al. 2018:281) at the hands of so-called diving pirates (Burchell 1971). Of particular interest in the postwar period is the apparent lack of official interest in relocating the site or introducing measures to, if not protect it, at least limit interference. This section brings together existing data with previously unpublished reports to establish a clear timeline of intervention (or lack thereof). I then turn to the joint survey in 2017—some 75 years after *Perth* sank—and the impetus it provided for Indonesia to introduce a maritime conservation zone around *Perth*. For some in Australia, however this was “too little, too late” (Wood 2017). Nevertheless, the logistics of how the zone is implemented present previously unexplored avenues for Australia to engage with Indonesia on the future management of *Perth* and, more significantly, create space for a broader conceptualization of who has a stake in this story. Whereas *Perth*’s value was once framed solely in terms of its historical and archaeological significance to Australia, the introduction of the maritime conservation zone serves to reintroduce Indonesia, not least of all the communities of Banten Bay, within the narrative. *Perth*’s material legacy has largely gone, but other considerations have surfaced: the marine ecosystems the wreck is home to, its potential to support sustainable livelihoods and tourism initiatives, and the connections it has with coastal communities. By recognizing *Perth*’s generative potential as an essential part of its legacy, I propose, a more inclusive and just approach for *Perth*’s communities may yet be achievable.

## Historical Background

**Fig. 1:**
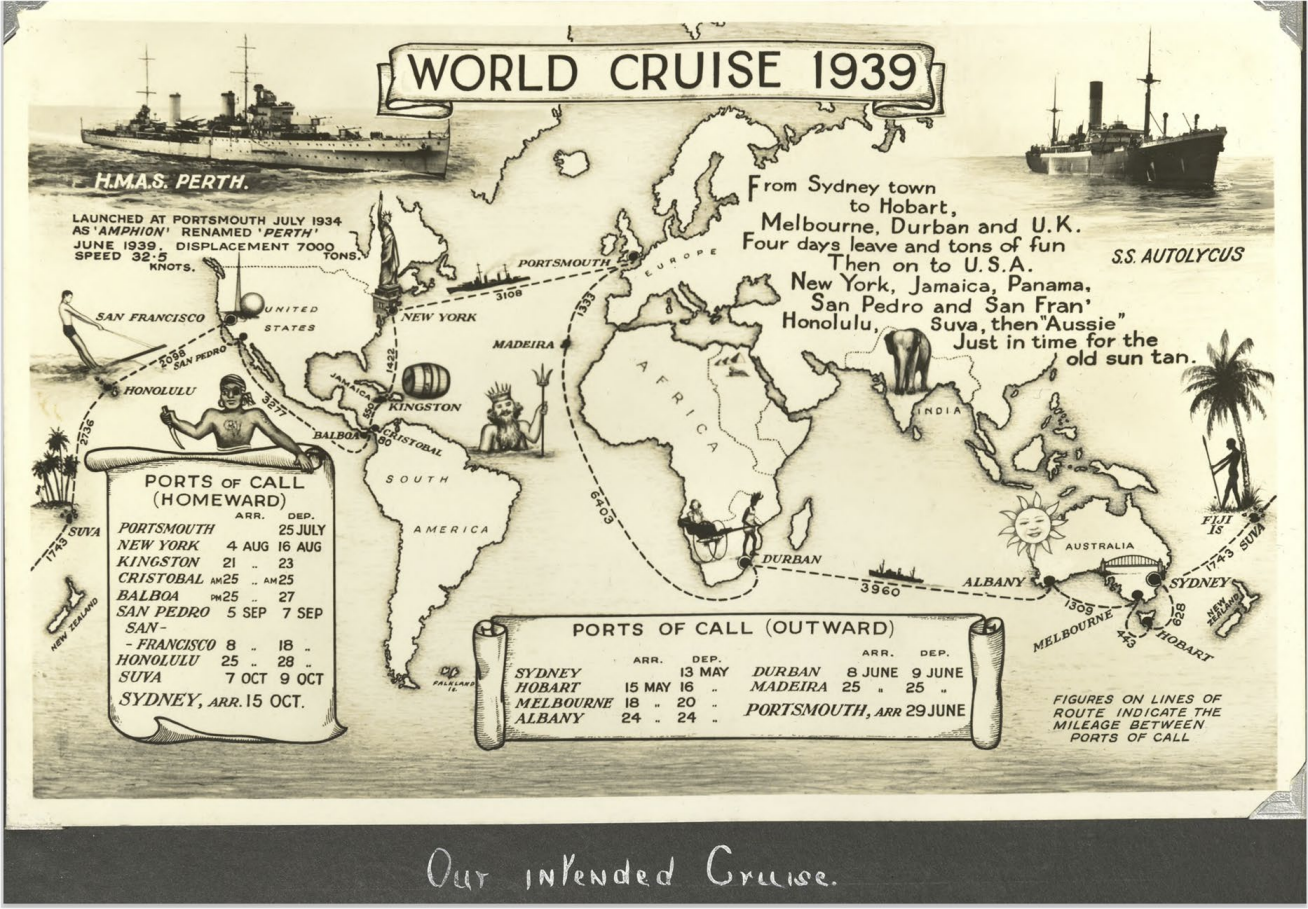
Postcard depicting *Perth*’s intended route to Australia, 1939 (Hatfield 2009).

**Fig. 2:**
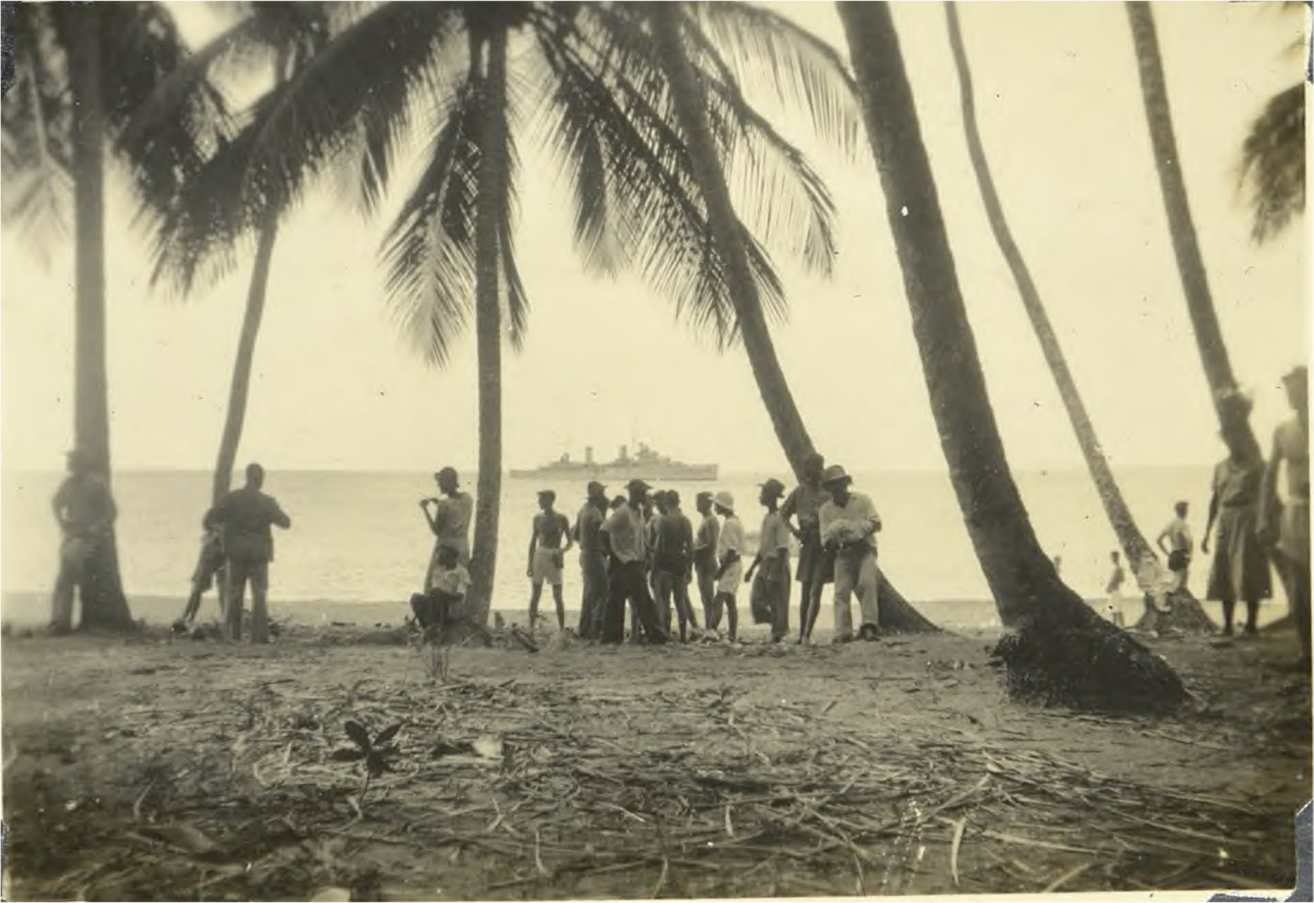
HMAS *Perth* (I) glimpsed through palm trees in Tobago (Hatfield 2009).

HMAS *Perth*, the first Royal Australian Navy ship to carry the name, was launched as *Amphion* in 1934. Built for the Royal Navy, it was one of three modified Leander-class cruisers later transferred to Australia. *Perth*’s early—and what was to be its only peacetime—service was a veritable “world cruise” marked by a sense of celebration (Hatfield 2009). Having traveled from Australia aboard SS *Autolycus* via the Indian Ocean and the west coast of Africa, *Perth*’s new crew were excited to enjoy “four days of leave and tons of fun” (Hatfield 2009:101) (Fig. 1) in England before joining their new ship in June 1939 and returning home via the Atlantic and Pacific oceans. However, the declaration of World War II disrupted these plans (Fig. 2), and it was not until March 1940 that *Perth* finally arrived in Port Jackson, Sydney.

After a refit and engine trials, *Perth* was tasked with what the sailors dubbed “ferry service” (Hatfield 2009:101)—patrol and escort work—around Australia, until November 1940 when it sailed for the Middle East and on to the Mediterranean. *Perth* arrived back in Australia in August 1941, where her crew were granted much-needed shore leave. A new commanding officer, Captain Hector Waller, was appointed and the ship underwent an extensive refit including the painting of a new, more angular, camouflage pattern.

As the situation to its north deteriorated, the Australian government responded favorably to a request for support from ABDA (American-British-Dutch-Australian) Command—an attempt, ultimately unsuccessful, by the Allies to run a combined military theater to Australia’s north. On 31 January 1942, *Perth* left Sydney for what was to be the last time. Arriving in the port of Tanjung Priok in Batavia (present-day Jakarta) on 24 February, *Perth* was directed to continue east to Surabaya where the ABDA fleet was concentrating for a last-ditch attempt to, if not prevent, at least delay the Japanese invasion of Java. On 27 February the two sides met in what came to be known as the Battle of the Java Sea. The engagement was an unmitigated disaster for the ABDA fleet (Manders et al. 2021).

*Perth* and *Houston* returned to Tanjung Priok, and from there continued west through the Sunda Strait. Despite having been advised the route was clear, they encountered the Japanese Western Invasion Convoy late on the evening of 28 February in waters near Banten Bay, west of Jakarta. The intense battle that ensued is known as the Battle of the Sunda Strait (28 February–1 March 1942). Both *Perth* and *Houston* were sunk by Japanese gunfire and torpedoes, with *Perth* sinking just after midnight and *Houston* shortly thereafter. Japanese ships were also damaged, with a transport and a minesweeper sunk (Underwater Archaeology Branch 2014; Remmelink 2015). *Perth* had 681 men embarked at the time, including naval personnel, air force personnel and civilian staff; of these, 353 died that night or shortly thereafter. Of the 1,061 in *Houston*, 693 were killed. Those who survived were taken as prisoners of war; many never made it home.

**Fig. 3:**
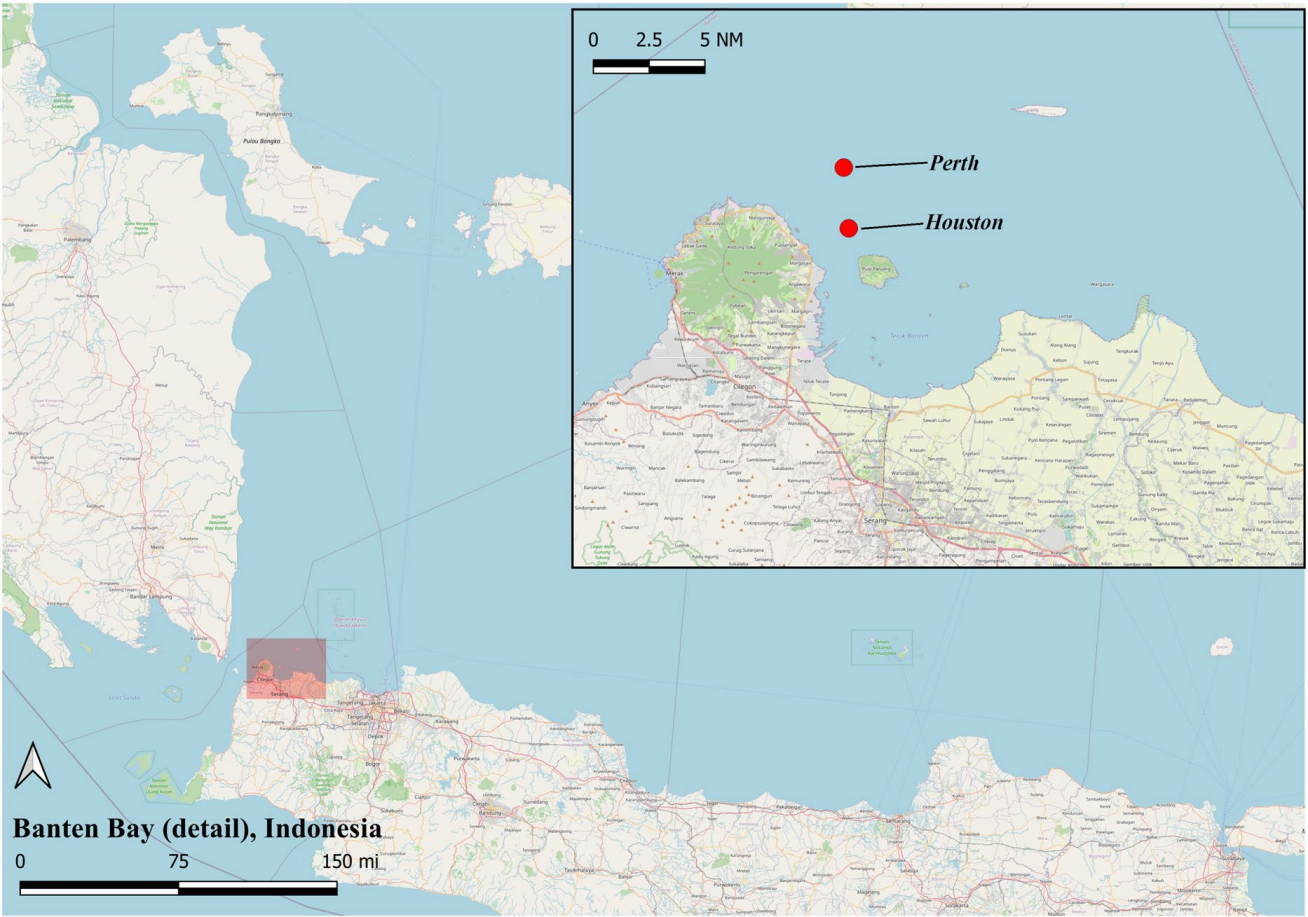
Map showing the Sunda Strait and the location of HMAS *Perth* (I) and USS *Houston* in Banten Bay. (Map created using QGIS by Natali Pearson [2022a, 2022b].)

The wreck of *Perth* settled on its port (left) side on the relatively flat, sandy seabed at a depth of approximately 115 ft. As illustrated in Figure 3, the site is about 3 nautical mi. northeast of St. Nicholas Point on Java’s northwest tip; the island of Pulau Panjang, at the entrance to Banten Bay, lies to the south-southeast. USS *Houston* sank about 2 nautical mi. south of *Perth* and therefore lies much closer to Pulau Panjang.

## The “Diving Pirates”

What could have been the beginning of a multilateral initiative involving Australia, the United States, and Indonesia to protect the wrecks and explore future management options was followed instead by decades of inertia. The lack of action to find and protect the wrecks should not be interpreted as a lack of interest or care in *Perth*’s fate, at least not in the immediate aftermath of the war. To a certain degree, the lack of effort in the postwar years to find *Perth* was symptomatic of the times. Following the end of World War II in 1945, many nations, including Australia and Indonesia, turned their attention to recovery and rebuilding; in the case of Indonesia, the postwar period was also characterized by a new era of independence for the former Dutch (and, from 1942 to 1945, Japanese) colony. Additionally, there was a strong sense—embodied in the sentiments of the “Naval Ode”—that sailors lost at sea were already in their final resting place: their grave was “the cruel sea,” their tombstone “a rusting hulk.”^1^ This was not about abandoning those who had lost their lives; it was a sign of respect for their identities as members of a ship’s crew, and an acknowledgment of the significance of their ship as home, battleground and eternal resting site. Finally, and not insignificantly, the technologies required to access a wreck at depth were still relatively nascent in the 1940s when *Perth* sank. As such, there was an assumption—albeit one that became outdated quite rapidly—that shipwrecks were implicitly protected by virtue of their inaccessibility. There was no need for the Australian government to protect *Perth* proactively or engage Indonesia; the ocean was already doing so.

It was not until 1967, some 25 years after *Perth* sank, that an attempt was made to locate the wreck. This was not an official expedition but one driven by an individual, Australian diver David Burchell, acting without institutional support (Burchell 1971). Concerned by newspaper reports that “diving pirates” were salvaging warships for scrap metal in the waters of Southeast Asia, Burchell took it upon himself to locate *Perth*—and if possible, given its proximity, *Houston*—and recover their bells, commonly considered to represent the soul of a ship, and deliver them to returned servicemen’s organizations (Burchell 1971:26). The regulatory frameworks relating to the management of shipwrecks were underdeveloped at that time both within Indonesia and internationally. In Indonesia, the only law pertaining to heritage was the colonial-era Monuments Ordnance dating to the 1930s and not yet updated post-independence. Its focus was on terrestrial monuments, and it made no provision for underwater sites, let alone sunken foreign warships. At the international level, none of the frameworks that now guide the management of heritage within territorial waters was yet established. Nevertheless, Burchell was cognizant of the potential sensitivities of searching for an Australian warship in Indonesian waters and took steps to advise both the Indonesian and Australian authorities of his intentions. When it eventually became clear that Burchell had indeed found *Perth*, the Australian Embassy in Jakarta, which had neither endorsed nor restricted his efforts, gifted him several tins of condensed milk—a tacit, but not quite formal, acknowledgement of Burchell’s success (Burchell 1971:97).

The presence of two large warships in Banten Bay was in fact common knowledge among local fishing communities, with Burchell having relied on members of these communities to help him find both *Perth* and *Houston* (*Daily Telegraph* 1967; Burchell 1971:59). The so-called “diving pirates” also knew of the wrecks’ locations. Throughout the late 1960s and into the early 1970s, Indonesian marine salvage firm PT Antasena conducted targeted salvage operations on *Perth* (Pearson 2017). The firm was headed by a former Indonesian general who oversaw the salvage of objects that were valuable, accessible, and portable, including the working and ceremonial bells, two phosphor bronze propellors from the starboard (right) side of the wreck (Pearson 2017; Hosty et al. 2018), and other nonferriferous items (Fock and Cannon 2013a, 2013b).

The absence of a proactive approach to locating *Perth* in the immediate postwar period is understandable. However, it is harder to account for the lack of action, on the part of both Australia, as flag state, and Indonesia, as coastal state, once Burchell had located *Perth*. Confirmation of the wreck’s location was a valuable opportunity for a new era of bilateral cooperation. However, despite bringing the matter to the attention of the Australian Embassy in Jakarta and actively engaging with former members of the Indonesian military, Burchell’s discovery of *Perth* did not result in any substantive policy changes. This was a lost opportunity that would come back to haunt both Australia and Indonesia.

Over time, *Perth* became an increasingly popular destination for divers. While increased visitation raised the risk of opportunistic souveniring by foreigners and locals alike (Hosty et al. 2018), it also enabled regular, albeit unofficial, monitoring of the site. The value of these recreational dives became evident when a group of technical divers noticed—and filmed, on 28 September 2013—unusual activity in the vicinity of the two aft (toward the stern of the ship) 6 in. gun turrets, including freshly torn metal, scoring marks and an absence of marine growth. This footage was provided to a small group of advocates who were able to compare it with their own footage from 2009. These advocates concluded that some of the damage observed in 2013 could be attributed to a period of accelerated natural collapse. Harder to explain was the presence on the seabed of items like steel cables (with no sign of corrosion or marine growth), gauges, boiler bricks, and a steel helmet. Although they acknowledged the cables may have been left behind by a snagged trawler, there was nevertheless sufficient evidence to suggest human interference on an ongoing basis. In the first of two reports provided to Royal Australian Navy officials, the advocates made three recommendations: a complete video survey of the wreck to determine the full extent of the collapse and whether salvage was occurring; an assessment of the risk posed by the presence of live ordnance; and recovery of any appropriate remaining artifacts for the Australian War Memorial (Fock and Cannon 2013a, 2013b).

Despite the discretion of the divers and advocates, who were committed to ascertaining the facts rather than spreading rumors, speculation mounted on the internet regarding the state of the wreck and the perceived lack of official action to protect it. The technical divers returned to the wreck site on 20 October 2013 to gather more data by inspecting the cables, the bow, and other areas of interest. Their findings, captured on video, were concerning: *Perth* was in the process of being extensively salvaged, most likely using a grab operating from a salvage barge. The nature of the damage to the forward part of the ship, including the removal of the forecastle deck and the gun houses of both forward turrets, was a clear sign of human interference. As such, the damage to the aft of the ship, which was earlier thought to be caused by natural collapse, was more likely caused by the grab. *Perth*’s advocates also noted with concern the probable desecration of human remains on the wreck site because of these salvage activities. In a second confidential report, dated 27 October 2013, the advocates warned that unless action was taken, it was “probable … the salvers will return and continue to pull apart the wreck, especially if their previous efforts have been remunerative” (Fock and Cannon 2013a, 2013b:24). Their recommendations pressed for urgent action to be taken to prevent further destruction of the wreck—but an official joint Australia-Indonesia dive to survey the wreck did not take place until May 2017. The delay was to prove devastating.

## Slow Steps toward Collaboration

Divers and advocates had raised the alarm within Australian government and military circles about human interference on the wreck, gathering historical data and digital footage to demonstrate the changes to the wreck over time. Despite their efforts, officials did not appear to take immediate action. It is difficult to account for this delay. Did legal and moral responsibility lie solely with one party, and was that party Australia or Indonesia? Could Australia have responded with more skill and agility, or was Indonesia to blame? Answering these questions requires consideration of the state of the bilateral relationship, an understanding of the complex bureaucratic process for foreigners seeking to conduct research in Indonesia, and knowledge of the (nonexistent) legal protection for *Perth*.

At the same time as these divers were sharing their concerns with the Department of Defence and other Australian authorities, relations between Australia and Indonesia were under severe strain. Media outlets were reporting that Australian intelligence agencies had tapped the phones of the president (Susilo Bambang Yudhoyono, 2004–2014), his wife, and other senior officials in 2009. In addition to recalling its ambassador to Australia, the refusal of then–Australian prime minister Tony Abbott (2013–2015) to apologize for what Abbott claimed were “reasonable” intelligence gathering activities prompted Indonesia to review the entirety of its bilateral cooperation activities (Griffiths 2013). When the *Perth* story broke publicly in December 2013, media outlets reported that Australian authorities had “tried to keep the scandal a secret, fearing the issue might add fuel to the ongoing diplomatic tensions between Australia and Indonesia” (Besser et al. 2016). Australia, having failed to proactively engage with Indonesia for decades, was paralyzed by the problems in the broader bilateral relationship and unable to respond rapidly to the threats *Perth* was facing. Indonesia, for its part, was perceived as “weak” (*lemah*) and ineffective for having failed to protect the wreck (Adhityatama 2014:84). Rather than being able to leverage an existing partnership, Australia and Indonesia had to establish a new collaboration from scratch.

The process took years. At the bilateral level, Australia’s chief of navy wrote to his Indonesian counterpart regarding the salvage works. Domestically, the RAN and the Commonwealth Department of the Environment (DoE, vested with responsibility for managing underwater cultural heritage in Australian waters) approached the Australian National Maritime Museum (ANMM) to lead an archaeological assessment of *Perth*. In December 2014, the ANMM hosted a visit to Sydney by representatives from DoE and researchers from the National Archaeological Research Agency (ARKENAS) to discuss and develop a memorandum of understanding (MoU) for the purpose of collaboration in research in maritime archaeology and underwater cultural heritage management. The MoU was signed by ARKENAS, ANMM, and DoE in August 2015, with an expiry date of three years from the commencement date. It provided an agreed framework for Australia and Indonesia to work together on maritime heritage, within which Indonesia wanted to focus on *Perth* due to staffing and resourcing considerations. While the MoU had a limited lifetime, it was, nevertheless, progress. In September 2015, as part of their site survey research proposal, the ANMM applied to Indonesia’s Ministry of Research and Technology (RISTEK) for a Foreign Research Permit.

As Australia and Indonesian inched their way toward formal collaboration, divers continued to visit *Perth*, as did salvagers. Some of these divers were state sanctioned; in June 2014, for instance, a joint team of divers from Indonesia’s Directorate of Cultural Heritage Preservation and Museums, ARKENAS, and Orca Diving visited the wreck to conduct an exploratory survey. Their report included an informant’s claim that salvagers had used explosives to blow up the ship’s bow (Adhityatama 2014). That same month, a team from the U.S. and Indonesian navies visited *Perth* briefly as part of a joint dive operation on *Houston*, which was also showing signs of interference (Underwater Archaeology Branch 2014). In January 2015, the Indonesian Navy (TNI-AL) apprehended a salvage barge above the wrecks of *Perth* and *Houston*, loaded with scrap metal, boiler tubes, and other components (Hosty et al. 2017). Two months later, in March 2015, four technical divers, operating in a private capacity, visited *Perth*. They recorded footage that showed signs of hull plate removal (Hosty et al. 2017:84). Footage taken in June 2015 by FROGdive DevGru, a dive group established by a member of the Indonesian Navy and open to the public, showed an “increased amount of observable salvage debris” (Hosty et al. 2017:85), most notably live ammunition. The U.S. and Indonesian navies again conducted a joint dive of *Perth* in October 2015, observing torn and buckled plating, damage to the forward gun housings, and numerous small artifacts scattered on the seabed. Also in October 2015, Indonesia’s Ministry of Marine Affairs and Fisheries, together with ARKENAS, conducted a side scan sonar survey of *Perth* that showed both aft, and one fore, gun turrets were still in place. Thus, between October 2013—when official reports had first been filed regarding suspected damage to *Perth*—and October 2015, the U.S. and Indonesia had managed to mobilize two joint surveys in Banten Bay, and representatives from Indonesian departments and agencies had also mounted four in-person or remote surveys. Meanwhile, Australia, following process, was still waiting on the outcome of their research proposal.

Finally, in April 2016, Australia’s proposal to collaborate with Indonesian researchers on *Perth* was approved. But further paperwork was still required. Whether Australia had failed to provide the necessary documentation in the first instance, or whether Indonesia was deliberately stalling, is unknown. Either way, the delay—and its consequences—were a salient reminder of the importance of including researchers with country- and culturally-specific knowledge, including language skills and familiarity with local bureaucratic procedures, in any research team working across jurisdictions. In the end, RISTEK did not issue a research permit and visa—essential for foreign researchers seeking to conduct research in Indonesia—to the ANMM’s team until October. This was too late to dive *Perth* in the optimum window between July and September, and the early arrival of the monsoon ruled out any chance at all that a joint Australia–Indonesia dive could take place in 2016. In the interim, ARKENAS and a private survey company were engaged in November 2016 to undertake a multi-beam sonar survey of the wreck. The survey revealed a number of anomalies, including a vast empty space amidships and a significant discrepancy in the apparent (295 ft.) and the original (561 ft.) length of the ship (Hosty et al. 2017:6).

At the same time as the multi-beam sonar survey of *Perth* was being organized in November 2016, another expedition, also relating to sunken allied warships, was taking place in Indonesian waters. In advance of the 75th anniversary of the Battle of the Java Sea (27 February 1942, in which both *Perth* and *Houston* had participated and survived), the Karel Doorman Fonds, a Dutch foundation that provides support to former navy personnel, had arranged for a group of divers to visit and survey the remains of Dutch and other allied wrecks in the Java Sea. According to Manders et al. (2021), the Dutch government had sought permission for the expedition from Indonesian authorities, but had not received a response. Because the divers had visited the sites previously, it was “assumed that no permit would be needed” for the dive or the associated documentary filming (Manders et al. 2021:42). The lack of permission became an “issue” when the divers not only realized that some of the wrecks in question had been entirely removed—presumed stolen—but brought up five items from the seabed, including some shell casings, as evidence they had been at the right location (Manders et al. 2021:43).

It was not until May 2017 that maritime archaeologists from the ANMM were able to survey *Perth* with their ARKENAS counterparts. After three-and-a-half years, Australia had finally established the formal engagement mechanisms and obtained the necessary permissions to undertake collaborative research with Indonesia. During this time, as their joint survey would reveal, the wreck of HMAS *Perth* had been destroyed, and any potential human remains desecrated, by deliberate acts of salvage on an industrial scale. Some 230 ft. of the ship’s stern was completely gone, from the stern post through to the aftermost engine room bulkhead, including four propeller shafts and the two aft gun turrets. The two forward guns and their turrets had been completely removed. Around 60% of the starboard hull plating was gone, leaving the vessel’s interior exposed. Internal compartments had been salvaged, including bulkheads, decks, fittings, steam turbines, condensers, and boilers. Both 4 in. shell magazines had been breached, with approximately 1,000 shells and cartridges removed; those that remained were leaching picric acid and highly unstable. The team noted stockpiles of copper and copper alloy cable and piping set aside for later recovery. There was also evidence of smaller scale salvage activities such as lifting slings, a chain block, and a hammer and chisel (Hosty et al. 2017:7–9).

*Perth* had experienced very little legal or regulatory protection in the decades it had spent underwater. As an Australian warship that had not been captured, expressly abandoned, or surrendered, *Perth* was—despite the ambiguous legal status of sunken state-owned vessels (Forrest 2003)—generally assumed to be the property of the Australian government. Had it sunk in Australian territorial waters, *Perth* could have been declared a historic shipwreck with an associated protected zone just as its sister ship, HMAS *Sydney* (II), had been upon discovery in 2008 (Mearns 2009). But the fact *Perth* lay beyond territorial waters eliminated Australia’s ability to protect it under domestic legislation. Nor was it recognized as an official war grave—such graves must be located in an officially designated cemetery with a headstone and administered by the Commonwealth War Graves Commission to be considered “official.” Indeed, the War Graves Commission does not even recognize the concept of maritime war graves (Forrest 2019:237–271).

Indonesia, meanwhile, had a checkered history with shipwreck management. In 1989 it had legalized the commercial salvage of valuable objects from certain historical shipwrecks, specifically “VOC [Dutch East India Company], Portuguese, Spanish, and World War II shipwrecks” in Indonesian territorial waters (Article 1 of Presidential Decision No. 43/1989) (Republic of Indonesia 1989). No activities were directed at *Perth* under this legislation; most salvagers working in Indonesia in the 1990s and 2000s were instead focused on finding and recovering valuable ceramic cargoes (Liebner 2014; Pearson 2022a). These commercial salvage activities effectively ceased in 2010 with the introduction of a new Cultural Heritage Law (Republic of Indonesia 2010) that opened the door for *Perth* to be designated as a cultural heritage site (to date Indonesia has inscribed just one underwater cultural heritage site under this law). Finally, neither Australia nor Indonesia are signatories to 2001 UNESCO Convention on the protection of underwater cultural heritage. Although the convention does not regulate the ownership of wrecks, it does provide for states parties to cooperate on underwater cultural heritage as well as explicitly stipulating that such heritage should not be commercially exploited (UNESCO 2001). The absence of laws to protect *Perth* was one factor behind the industrial scale salvaging; the other factor was its economic value.

The presence of valuable low background steel, as it is known, is commonly invoked as to why warships like *Perth* have been targeted for salvage (Holmes et al. 2017; Manders et al. 2021). This is steel manufactured prior to 1945, when the first nuclear weapon was detonated, and therefore not exposed to the radiation that has permeated the earth’s atmosphere ever since. Warships built and sunk before 1945 have been protected from exposure to atmospheric radiation, making them rare assemblages of a finite commodity. Low background steel is considered to be highly attractive to salvagers due to its value on the market for technologies such as Geiger counters and space and medical equipment (Manders et al. 2021). However, some experts are skeptical that low background steel is the driving force behind the illicit salvaging of *Perth* and other warships. The uses of low background steel are minute, and global demand for such steel is not high. Furthermore, atmospheric radiation has decreased significantly since the cessation of aboveground nuclear testing, meaning it is again possible to produce steel with a low radioactive signature. While the low background steel angle adds a level of intrigue to the wider story of stolen warships, it should be treated with a degree of caution. The more likely explanation as to why warships have been targeted is more prosaic: salvage barges such as the one seen above *Perth* in January 2015 were already operating legally in the region’s waters to clear sea lanes (Simorangkir et al. 2020). These operators did not need to mount an expedition from scratch: the barge and the crew were already engaged, and the only additional cost was the fuel. Salvaging of the illicit kind was simply a low-cost, high-profit, extracurricular addition to their other, legal, activities.

There were, therefore, many factors contributing to *Perth*’s vulnerability. Foremost among these were Australia’s failure to proactively engage with Indonesia, the complexity of obtaining formal research permission accompanied by a lack of understanding about what was needed to navigate this bureaucracy, an absence of effective legal protection, and the presence of salvagers in the region who knew the value of scrap metal (Vercammen et al. 2017). As determined by the 2017 joint survey, these factors combined to result in the destruction of the wreck and the human remains it was home to. This was a devastating blow for the divers and advocates who had first raised the alarm in 2013, as well as for *Perth*’s communities of survivors and descendants. Online, *Perth*-related forums lit up with criticism:

It is highly insulting that a war grave is being degraded. The dead are being distributed for utter greed. I bet if it was their [Indonesia’s] war graves being disturb [*sic*] their screams would be heard from Djakarta.

Rather than destabilizing the bilateral relationship, however, the survey results galvanized both Indonesia and Australia to take steps to protect the little that remains, in the process creating a new legacy for *Perth*.

## Building Legacy through Community

HMAS *Perth* was, for many years, defined by its connection to Australia. It was part of Indonesia’s story only insofar as that was where *Perth*’s final battle had taken place. The industrial scale salvaging of *Perth* was devastating for survivors and descendants not only because of the material destruction and human desecration that had occurred, but because of what this meant for *Perth*’s legacy. Rather than the story of a proud ship and its crew “afast on the ocean bed,” was *Perth*’s legacy instead to be one of men abandoned by their government and of historical and archaeological value overlooked in favor of profit? Was a legacy even possible with so much of the wreck gone? Rather than being defined by loss, however, a new legacy for *Perth* has emerged since the results of the 2017 survey became public. The results galvanized the Australian and Indonesian governments into action, with both focusing on *Perth* in a way that neither had in the past. The effect was a broadening of *Perth*’s legacy, through a deepening of its connections to the communities and histories of Banten Bay.

On the symbolic date of 28 February 2018, to coincide with the 76th anniversary of the Battle of the Sunda Strait, Indonesia’s Minister of Marine Affairs and Fisheries, Susi Pudjiastuti, issued a ministerial decree (Republic of Indonesia 2018) that designated HMAS *Perth* as a maritime conservation zone (*kawasan konservasi maritim*). The designation was prompted by the findings of the 2017 ANMM/ARKENAS survey and report, and followed heavy lobbying by representatives in both the Australian and Indonesian governments. The maritime conservation zone provides a legal basis for the ongoing protection and care of the site and represents a major shift in the way both Indonesia and Australia have approached *Perth*. Instead of shying away from the complexities and sensitivities of managing an Australian warship in Indonesian waters, as had been the case since 1942, the establishment of the maritime conservation zone was a sign that both countries were committed to ensuring better management outcomes for the wreck into the future—and to doing so together.

Despite the provisions in the 2010 Cultural Heritage Law, this was not a cultural heritage, but a maritime conservation, zone—Indonesia’s first such zone. This decision recognized that cultural and natural values coexisted at the wreck, thereby aligning with the provisions of an earlier ministerial regulation (Republic of Indonesia 2008) that characterized maritime conservation zones as sites of historical and archaeological significance as well as places that hold natural values for coastal and small island communities. By protecting *Perth* and its marine ecosystem, the Indonesian government was able to pursue two outcomes—preservation of the wreck and the conservation of marine life—with one legislative stroke. In doing so, it sought to both guarantee the sustainable management of the wreck as underwater cultural heritage as well as improve the welfare of the coastal communities—such as those on Pulau Panjang, as well as the subdistricts of Argawana, Pulo Ampel, and Salira—in and around Banten Bay.

**Fig. 4:**
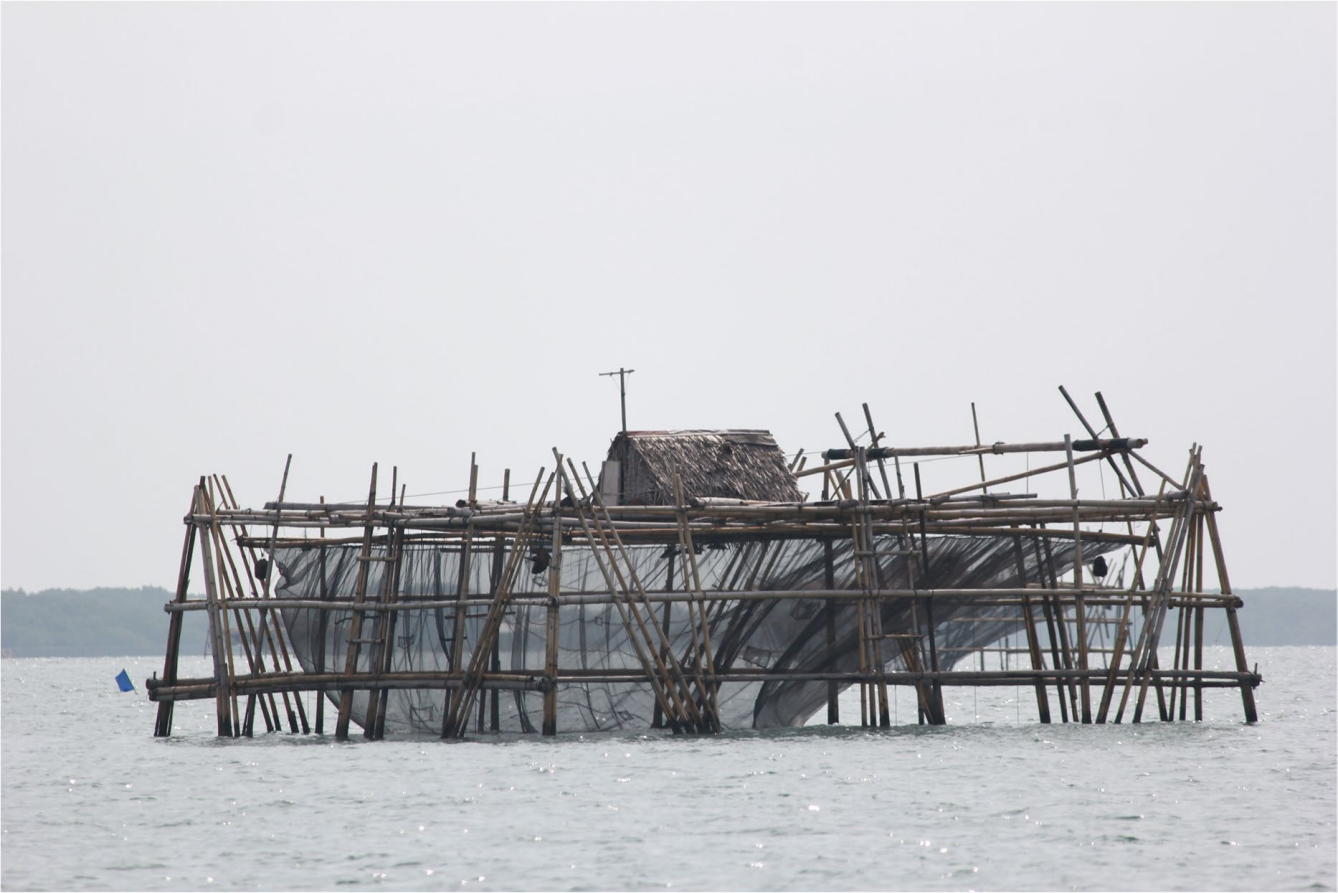
Floating net cages in Banten Bay. In addition to being located close to a busy industrial port, the marine space around HMAS *Perth* (I) and USS *Houston* is in heavy use by those who make a living from fishing and aquaculture, including anchovies and lobsters. (Photo provided to the author by personal correspondence.)

Although the ministerial decree had been designated by national authorities, responsibility for managing maritime conservation zones lies at the provincial level. This provision is designed to enable a level of implementation and surveillance that would not be possible if authority were centralized in the Ministry of Marine Affairs and Fisheries. Following *Perth*’s designation, officials from the Office of Marine Affairs and Fisheries, Banten Province, were given funding to develop a plan to manage *Perth* for the future, which they did in consultation with other provincial government agencies including the offices of Tourism, Education and Transportation. The management and zoning plan they developed seeks to balance the significant responsibility of managing *Perth* with the needs of the local community by providing for two zones: a core zone and a zone of limited use, with a total area of 99.94 hectares. The core zone is the 9180 m^2^ area of the wreck itself and the water column above it. Only limited activities are permitted within the core zone: surveillance patrols, research and non-extractive development, and educational activities. The larger zone of limited use permits a wider range of activities for the benefit of local communities, including pilgrimages or religious ceremonies, water-based tourism, fishing, and aquaculture (Fig. 4).

**Fig. 5:**
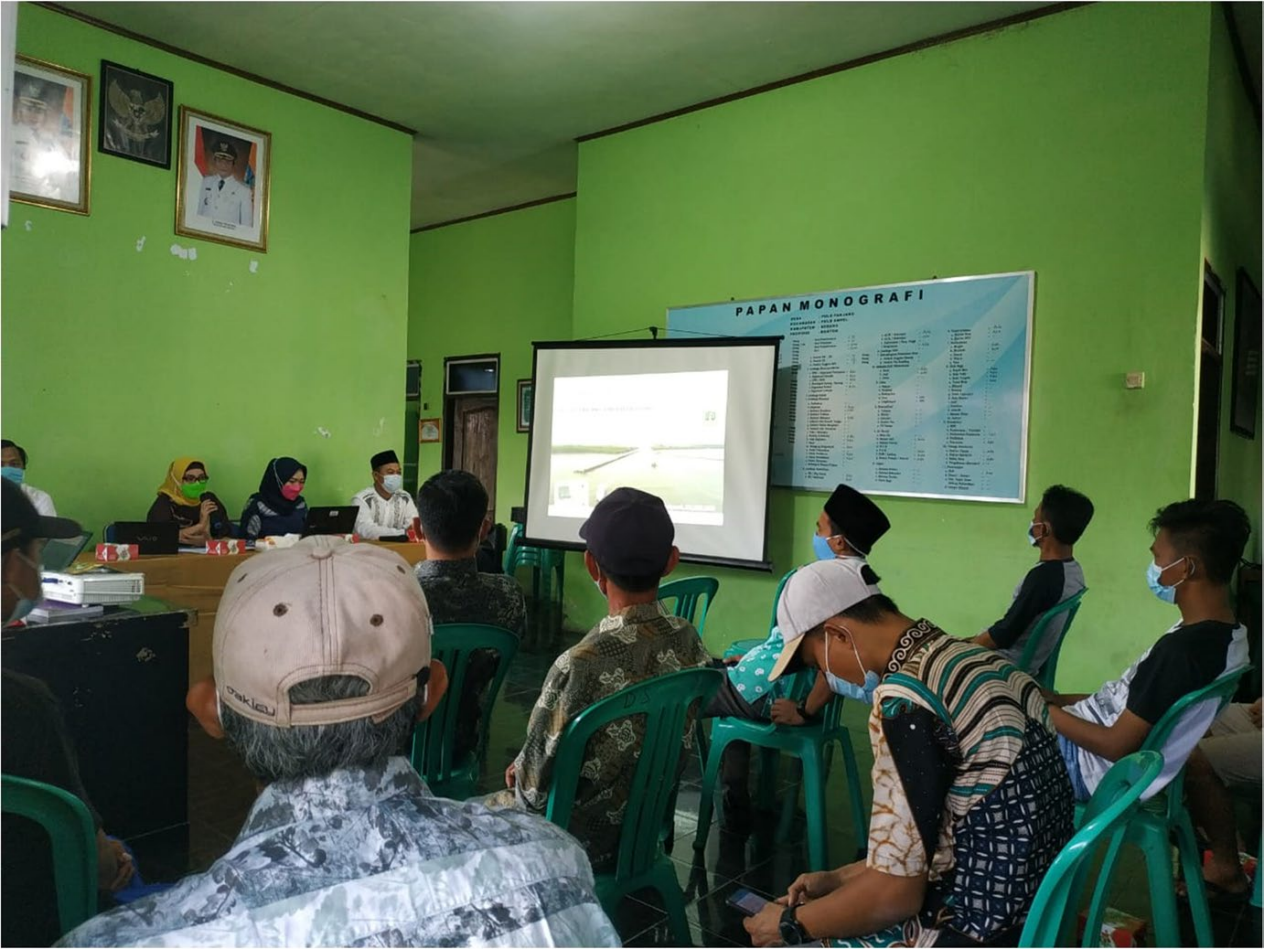
Socialization and awareness raising session with residents of Pulau Panjang about the conservation area management for coastal and small islands. (Photo provided to the author by personal correspondence.)

The plan provides for monitoring and surveillance through routine and integrated patrols conducted with representatives from the police, navy, and Ministry of Marine Affairs and Fisheries. Authorities are also working with a community-based group of “guardians” (*Kelompok Masyarakat Pengawas*, or *Pokmaswas*) based in Pulau Panjang who play a key role supervising the site when authorities are not available. These local guardians live and work, as anchovy and lobster fishers, close to the wreck site. As such, supervising *Perth* is easily integrated into their daily activities. While security is a priority, the plan also focuses on developing capacity within the local community. In the short term, this includes raising awareness about HMAS *Perth* and its historical significance, thereby creating opportunities for locals to foster a deeper connection to the wreck(s) in their waters (Fig. 5). Officials are also keen to develop human resource skills by training community members to act as guides or produce handicrafts related to *Perth*. In the longer term (2019–2038), specialist dive tourism is a possibility, although it would be tightly regulated by the provisions of the management and zoning plan (Pearson 2022b). The “big dream” (Perth’s Stories 2021b), however, is to build an information center or even museum that portrays the Battle of the Sunda Strait and provides context about the marine ecosystems of Banten Bay. As local fisheries official Risnawati Rahayu explains,

We hope that such an information centre will contribute to the welfare of the society. With the existence of an information centre, we will get educational benefits, cultural benefits, and ecological benefits. Visitors to the Centre may ... take a boat tour, circling above the shipwreck [using] boats owned by fishermen living around the site of HMAS *Perth*. And I think this will have a positive impact economically on the local communities. So, the information centre will provide various benefits to the communities. (Perth’s Stories 2021b)

These short- and long-term activities contribute to what Tahir (2014:822) describes as a sense of “community wellbeing,” whereby people develop a sense of the benefits, not just the responsibilities, of living with a historically significant wreck in their waters. This observation aligns with the sense of pride (“*sangat bangga*”) expressed by Pak Wahyu Iwan Setiawan, the village chief of Pulau Panjang village, regarding “the existence of historical objects, namely World War II wreckage,” of which he had been aware since he was a child (Perth’s Stories 2021a). In this conceptualization, heritage, local livelihoods, and ecosystem preservation are closely connected, with shipwrecks such as *Perth* playing a central role in this relationship. Such initiatives represent a more sustainable solution to the question of how to best manage *Perth* for the future and stand in stark contrast to the short-term exploitation that has taken place at the hands of illicit salvagers.

Greater engagement with local communities in Indonesia has exposed sensitivities within veterans’ groups in Australia. In 2022, media reporting that *Perth* was to be opened to marine tourism and diving prompted fury in some circles. In Australia, military historian Pattie Wright argued *Perth*

should not be turned into anything even close to a tourism site. The Indonesian side has not protected any of the wreck sites in their waters. They should not now be able to make money on what is left. This is a sacred site of officers and men who died fighting for our country. Leave it alone. (Barrett and Rompies 2022)

The proposal was also met with resistance in the United States. John Schwarz, executive director of the USS *Houston* Survivors’ Association and Next Generations, said:

On just [*sic*] the night alone when they were attacked and sunk together, within a total of about 50 minutes, over 1000 servicemen lost their lives. And someone wants to make their burial ground a tourist attraction? (Barrett and Rompies 2022)

Similar sentiments were evident in comments posted on online *Perth*-related forums. As Perth’s legacy shifts and expands, addressing the concerns of communities in both Indonesia and Australia will be of critical importance.

## New Partnerships for the Future

Representatives from Australia and Indonesia have continued to collaborate on maritime heritage activities. In May 2019, archaeologists from ANMM and ARKENAS came together a second time to conduct a joint dive (Hosty et al. 2020). This activity was supported through the Maritime Capacity Building Initiative (MCBI), funded through the Department of Foreign Affairs and Trade (DFAT) for a project on “HMAS *Perth* (I) Wreck: Preservation and development of tourism potential.” The project—designed pre-pandemic and funded, at least initially, from December 2018 to July 2020—provided for not only the second joint diving expedition, but for the engagement of a heritage adviser to undertake stakeholder consultations and develop costed management options that balanced site preservation with the creation of economic opportunities for coastal communities.

However, the travel restrictions and other disruptions caused by the COVID-19 pandemic from early 2020 onward made in-country stakeholder engagement activities unviable. In lieu of face-to-face consultations, the project shifted to focus on building understanding at different levels—national, provincial, local—within Indonesia about the presence and preservation of *Perth*. To this end, the project team ran a series of online Historic Shipwreck Management workshops in November 2020 with government stakeholders, academics and diving practitioners about the significance of *Perth* and the need to work together to protect it. The workshops were attended by representatives from the ministries of Marine Affairs and Fisheries, Education and Culture, Foreign Affairs, Defence and Navigation, as well as the Indonesian Navy and provincial level officials, and focused on issues relating to maritime conservation zones, sustainable management of World War II wrecks, and public education and engagement. The final module was designed to facilitate discussion between Indonesian and Australian stakeholders and formulate a way forward for the management of *Perth*. Although different from the consultation originally envisioned under the MCBI, these workshops allowed Australian researchers to identify key players and build relationships.

As became evident during the workshops, one of the major stakeholders for *Perth*-related consultations is the Office of Marine Affairs and Fisheries, Banten Province. During the Historic Shipwreck Management workshops, fisheries officials shared their vision to develop a museum that positions Banten as an international pilgrimage destination. They noted that the wider Banten region holds spiritual significance, with sites in the Banten Lama district—including the Great Mosque and the Kasunyatan Mosque, as well as several royal sepulchers—associated with the historic sultanate dating to the 16th and 17th centuries. Additionally, the bay itself is home to seagrass fields, coral reefs, and mangrove areas, which support a variety of important wildlife including the Irrawaddy dolphin as well as commercial fish species that serve as an important economic base for local communities. These religious and environmental aspects, the officials proposed, could be woven together with the historical considerations associated with *Perth’s* and *Houston*’s presence to create a unique tourist destination with international appeal.

**Fig. 6:**
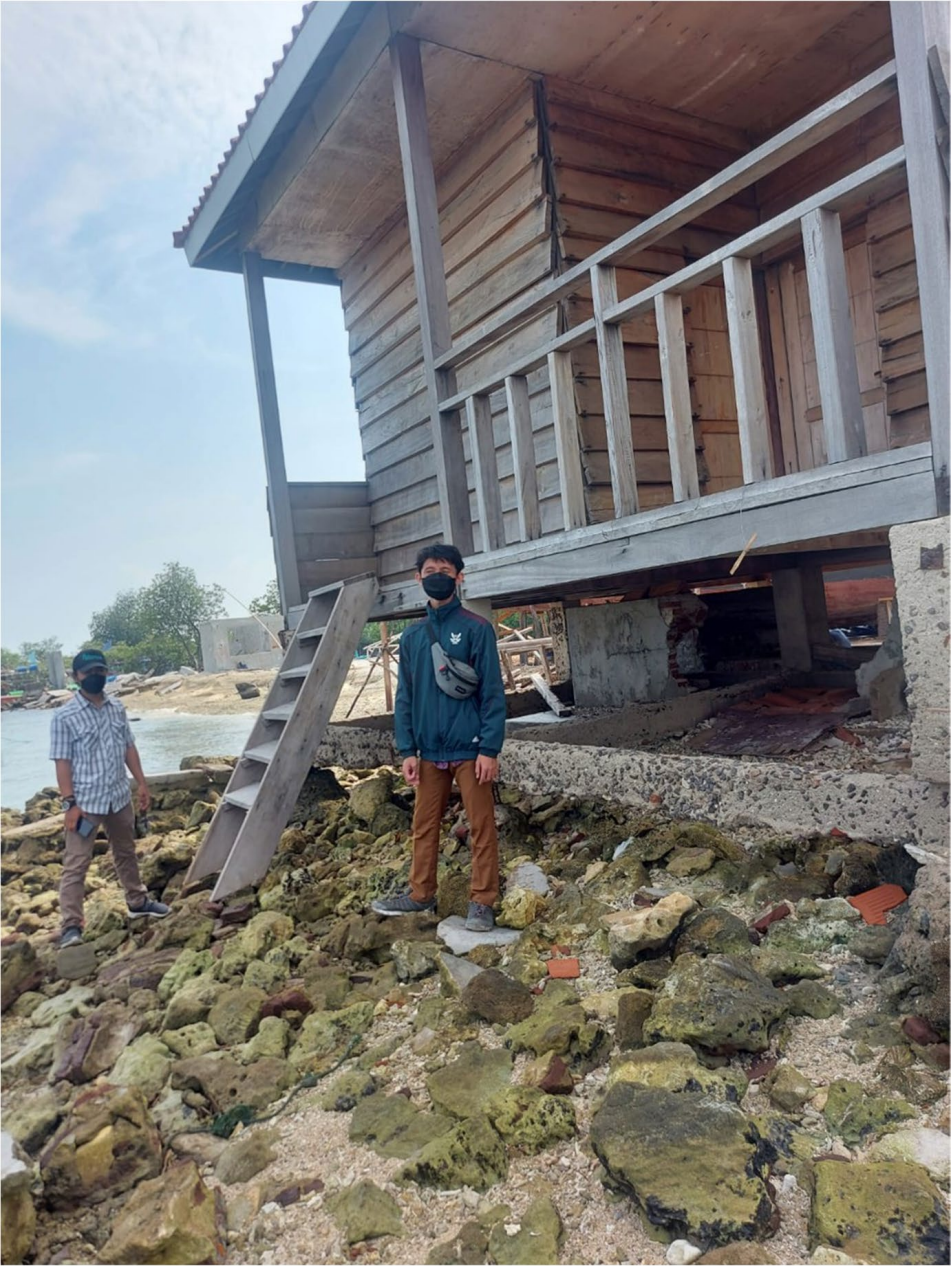
Guardhouse built on Pulau Panjang in 2019 but not yet operational due to lack of funding. (Photo provided to the author by personal correspondence.)

These officials had led the preparation of the management and zoning plan, which, despite being drafted in August 2018, has still not been fully implemented or funded because it has not been approved at the provincial level. Such approval is understood to be contingent on Australia and Indonesia establishing a more strategic and sustainable model for future engagement through a new agreement that would embed bilateral maritime heritage collaboration within Australia’s broader heritage management program. Negotiations on this agreement have been ongoing for several years and were further delayed in 2021 following a machinery of government change that saw ARKENAS moved out of Indonesia’s Ministry of Education and Culture and into the new National Research and Innovation Body (Badan Riset dan Inovasi, or BRIN) (Burhani et al. 2021). Until this agreement is finalized, it is considered unlikely the Management and Zoning Plan for HMAS *Perth* will be approved and funded. This situation, which remains ongoing, is a direct result of Australia’s failure to engage with Indonesia on *Perth* for many decades. Despite the lack of funding, however, local officials have been able to incorporate monitoring and surveillance of *Perth* into their existing work activities. Other aspects, such as the development of a site interpretation center or the operationalization of a guard house on Pulau Panjang, remain unfunded (Fig. 6).

To ensure access to knowledge for ongoing capacity building, the MCBI project team also developed a limited podcast series, *Perth’s Stories,* available on Soundcloud (University of Sydney 2022). This series features 10 interviews with 13 people, each of whom have a close connection with *Perth*. They include survivors, descendants, historians, advocates, officials, scholars, archaeologists, and Banten Bay locals. Podcasts were recorded in English or Indonesian in accordance with the interviewee’s preference, with bilingual transcripts available to promote greater access (University of Sydney 2022).

Building on new partnerships developed through the MCBI, a collaborative team from the University of Sydney (Australia) and the Ministry of Marine Affairs and Fisheries (Indonesia) came together as part of a new project in 2021 to co-design and deliver an online short course for Indonesian undergraduate students. The project, Collaborative Approaches to Capacity Building in Indonesian Maritime Archaeology, was funded by the Australia-Indonesia Institute at DFAT (Pearson and Tahir 2021). It brought together 17 students of archaeology and education from across the country, including from Universitas Indonesia (UI, Jakarta), Universitas Gadjah Mada (UGM, Yogyakarta), Universitas Hasanuddin (UNHAS, Makassar), Universitas Jambi and, in recognition of its proximity to the *Perth* wreck site and local communities in Banten Province, Universitas Sultan Ageng Tirtayasa (UNTIRTA, Serang).

Students developed a deeper understanding of the challenges and opportunities of managing maritime heritage in an Indonesian context, and established new networks across geographies, generations, and institutions. A central feature of the program was the student-led research project, in which students worked in small cross-institutional groups of 4–5 members to develop a research project on an issue relating to maritime heritage in Indonesia. In an indication of the importance of engaging with coastal communities, one of these small research groups developed a proposal to mount a temporary exhibition on HMAS *Perth* in the UNTIRTA auditorium. They identified a number of salvaged objects for display, including *Perth*’s two bells, as well as a 3-D visualization of *Perth* inspired by Curtin HIVE’s Sydney-Kormoran Project (Woods 2019). Three of the UNTIRTA students were later interviewed for the Perth’s Stories podcast (Perth’s Stories 2021c).

The Collaborative Approaches to Capacity Building in Indonesian Maritime Archaeology Project was designed to align with the priorities and objectives of the 2018 bilateral Joint Declaration on a Comprehensive Strategic Partnership (CSP) between Australia and Indonesia that identifies effective and meaningful maritime engagement as one of five broad “pillars” of cooperation (Australian Government 2018). In particular, the CSP identifies the need to strengthen educational and academic cooperation, encourage exchanges across institutions, and promote maritime cultural heritage. The project thereby created a new pathway for engagement between Australian researchers and the next generation of Indonesian maritime heritage practitioners and archaeologists that aligned with the strategic objectives of both countries. Not only did it enable Australia and Indonesia to strengthen maritime heritage cooperation—a priority for both countries—but the online format also filled a gap created by pandemic-related travel restrictions. In doing so, the partners were able to consolidate their nascent collaborative relationships while also investing in new relationships with students and institutions across the archipelago.

Project team members from Australia and Indonesia have subsequently collaborated to deliver an online learning session for the Marine Heritage Gallery in Jakarta—held on 1 March 2022 on the 80th anniversary of *Perth*’s loss—for high-school students (Marine Heritage Gallery 2022). They have also presented at international public events, including on the links between local tourism and marine heritage protection in an Indonesian context (Christian Michelsen Institute and University of Bergen 2022), and at area studies and archaeology conferences in Australia. Although the COVID-19 pandemic interrupted plans to conduct community consultations with people living in and around Banten Bay, new and productive collaborations have arisen that will provide valuable ground for future initiatives. Finally—and even though much work remains to be done to ensure the management plan is signed and to engage local communities in ways that are mutually beneficial—Australia and Indonesia are working together on *Perth*.

## Conclusion

In World War II, and again in the 2010s, the story of HMAS *Perth* has been defined by loss. The grief that permeates Australian survivor and descendant groups about the sorry fate of *Perth* and its crew is both profound and justified. But the dynamism of *Perth*’s legacy—which is now being understood in different ways and implicating new communities—points to the possibility of a more sustainable model for managing underwater cultural heritage. It also suggests a new way of thinking about and with wrecks, in a way that “humbly negotiates” with the water rather than seeking “eternal preservation” through deliverance from the sea (Rich 2021:21–22).

One of *Perth*’s emerging legacies is the lesson it offers for Australia and other flag states about the need to proactively engage with coastal states to manage wartime maritime heritage. *Perth* is just one of a number of WWII vessels lost in or near Indonesian waters; there is also HMAS *Yarra* (II) (lost in action, 4 March 1942, well off Java’s south coast) and the *Mustika* junk (scuttled by Force Z, October 1944, near Batam), while HMAS *Armidale* (I) (sunk by Japanese aircraft, 1 December 1942) is slightly farther afield off Timor’s south coast. And, while the establishment of a formal agreement for bilateral maritime heritage engagement will represent an important step at the government-to-government level, it is not a necessary precursor to the ongoing work of relationship building between people and between institutions. *Perth* could also be used to lobby the Commonwealth War Graves Commission to officially recognize maritime war graves or the opportunity to educate the public on the memorialization of sailors lost at sea in conflict. As a case study of a threatened shipwreck, *Perth* offers salient lessons—for maritime archaeologists, diplomats, and heritage practitioners—on the need for nations to engage proactively and sensitively around shared naval heritage and of the consequences of failing to do so. It also draws attention to the role of recreational and specialist divers within a shipwreck’s community and the value of their care through regular visits and informal site monitoring.

Equally valuable is the extent to which *Perth*’s legacy has expanded to include local communities in Banten Bay. The designation of *Perth* as a maritime conservation zone created a legally enforceable basis for protecting the wreck; importantly, it did so by attending to the potential benefits for the coastal communities of Banten Bay. Recognizing the connection between *Perth* and local livelihoods is not about exploiting the wreck by making a tourist attraction of a burial ground (Barrett and Rompies 2022). Nor is it about community members policing the wreck on behalf of foreign governments. Instead, it is about raising awareness of the role of *Perth* within Indonesia’s own history, if not through its role in resisting Japanese invasion, then as a material presence in local waters for eight decades. A more inclusive approach to conceptualizing *Perth*’s communities need not come at the cost of the wreck’s protection and preservation; in fact, such an approach is more likely to lead to more sustainable management outcomes as communities develop and define their own relationship with the wreck. USS *Houston*, which is still largely intact and for which designation as a maritime conservation zone is imminent, also offers important opportunities in this regard.

*Perth*’s sad fate challenges us because it requires us to reckon with loss on a large and endlessly repeating scale. As Rich writes, humans, animals, objects, and other “active participants of Earth” grapple with mortality and loss on a daily basis (Rich 2021:23). These themes—of loss and destruction and the counterbalance promised by efforts to protect and preserve—are not uncommon in maritime archaeology. Indeed, the study of shipwrecks is heavily reliant on the tragedies that occur at sea. Where *Perth* does test theoretical limits, however, is in the recurring and compounding nature of this loss, and the ways in which communities, broadly defined, as well as nations, have been forced to reckon with it. The extent to which this reckoning is destructive, as it has been, or generative, as it has the potential to be, is now the defining question. To answer this, *Perth*’s communities—from the governments charged with protecting it, to the people who care about it—must sit with the knowledge that not enough was done to protect *Perth* from the violence of indiscriminate salvage. Perhaps, in the end, *Perth*’s most poignant legacy is the lessons it holds about the potential for destruction to be productive when it inspires a broadening of the community of stakeholders. In this regard, *Perth* is not just an Australian story, or an Indonesian one, but a global one with profound relevance to other sites of meaning and loss.

## Conflict of Interest

The author reports there are no conflicts of interest.

## Footnotes

1 “They have no grave but the cruel sea, No flowers lay at their head, A rusting hulk is their tombstone, Afast on the ocean bed” (Driftwood 2023).
